# A Tracing Model for the Evolutionary Equilibrium of Octoploids

**DOI:** 10.3389/fgene.2021.794907

**Published:** 2022-01-28

**Authors:** Jing Wang, Xuemin Lv, Li Feng, Ang Dong, Dan Liang, Rongling Wu

**Affiliations:** ^1^ National Engineering Laboratory for Tree Breeding, Key Laboratory of Genetics and Breeding in Forest Trees and Ornamental Plants, Ministry of Education, The Tree and Ornamental Plant Breeding and Biotechnology Laboratory of National Forestry and Grassland Administration, Center for Computational Biology, College of Biological Sciences and Technology, Beijing Forestry University, Beijing, China; ^2^ Departments of Public Health Sciences and Statistics, Center for Statistical Genetics, The Pennsylvania State University, Hershey, PA, United States

**Keywords:** Hardy-Weinberg equilibrium, polyploid, natural population, EM algorithm, switchgrass

## Abstract

Testing Hardy-Weinberg equilibrium (HWE) is a fundamental approach for inferring population diversity and evolution, but its application to octoploids containing eight chromosome sets has not well been justified. We derive a mathematical model to trace how genotype frequencies transmit from parental to offspring generations in the natural populations of autooctoploids. We find that octoploids, including autooctolpoids undergoing double reduction, attach asymptotic HWE (aHWE) after 15 generations of random mating, in a contrast to diploids where one generation can assure exact equilibrium and, also, different from tetraploids that use 5 generations to reach aHWE. We develop a statistical procedure for testing aHWE in octoploids and apply it to analyze a real data set from octoploid switchgrass distributed in two ecologically different regions, demonstrating the usefulness of the test procedure. Our model provides a tool for studying the population genetic diversity of octoploids, inferring their evolutionary history, and identifying the ecological relationship of octoploid-genome structure with environmental adaptation.

## Introduction

As an evolutionary force of the organism to buffer against environmental perturbations, the evolutionary mechanisms of polyploidy have been a long-standing subject of population and evolutionary genetic research (Bever and Felber 1992; Ramsey and Schemske 1998; Otto and Whitton 2000; Soltis et al., 2004; Fawcett et al., 2009). While a number of population genetic studies are focused on tetraploids (Haldane 1930; Moody et al., 1993; Butruille and Boiteux 2000; Arnold et al., 2012; Dufresne et al., 2014; Meirmans et al., 2018; Sun et al., 2020), there is little knowledge about population variation in polyploids at a higher ploidy level. Octoploids, whose cells have a chromosome number as many as eight times the basic haploid chromosome number, are an even less-explored group of polyploids (Edger et al., 2019). As compared to tetraploids, octoploids have wide genetic diversity that can better adapt to changing environment (Johnson and Vance-Borland, 2016; Grabowski et al., 2017). Also, octoploids may have a larger body or organ size than tetraploids, implying their more desirable opportunity to be used in breeding programs. For example, basin wildrye have larger leaves, longer culms, and greater crown circumference for octoploids than tetraploids, although the numerical ranges of plant traits and their source climates overlap between ploidy types (Johnson and Vance-Borland, 2016). By creating a ploidy series from tetraploids to octoploid for althea (Hibiscus syriacus), Lattier et al. (2019) found an increase in guard cell length and rDNA signals with the level of ploidy.

Despite their evolutionary and economic value, it is unclear how octoploids vary and evolve across time and space scales, largely owing to the lack of suitable population genetic analysis tools. Hardy-Weinberg equilibrium (HWE) test has been widely used as an approach for inferring the evolutionary processes of natural populations (Waples 2015), but this approach was established on diploids, leaving its use to polyploids a mystery. Although the diploid-driven HWE test procedure has been modified to accommodate tetraploids (Moody et al., 1993; Meirmans et al., 2018; Sun et al., 2020), the use of such a modification to evaluate population variation in inherently more complicated octoploids is not justified. For example, double reduction has little impact on the asymptotic process of HWE for autotetraploids, but we do not know if this is true for autooctoploids that produce three types of gametes, characterized by two, one, or no double reduction, respectively. Sun et al. (2020) proposed a gamete-based approach for testing autotetraploid aHWE by estimating three equilibrium diploid gamete frequencies. This approach has a power to test the equilibrium of dosage-unknown markers because three gamete frequencies just can be estimated by three observable types of genotypes. However, this approach has no sufficient degrees of freedom to test aHWE for dosage-unknown markers in autooctoploids that produce five tetraploid gametes but still have three observable types of genotypes.

In this article, we propose an approach for HWE testing in octoploids using any type of molecular markers. We derive a system of recursive equations that transmit individual octoploid genotype frequencies from the parental to offspring generation under random mating. We find that, as opposed to diploids in which exact equilibrium can be reached after one generation of random mating, both allooctoploids and autooctoploids can only gradually approach aHWE after 15 generations, different from tetraploids that use 5 generations to reach aHWE. We propose specific statistical procedures for testing aHWE using dosage-known and dosage-unknown markers in octoploids. We investigate the power of octoploid HWE detection through computer simulation. By analyzing population genetic data of allooctoploid switchgrass (Grabowski et al., 2017), we validate the usefulness of our testing procedure.

### Mathematical Model

Our analysis is based on the segregation of biallelic single nucleotide polymorphisms (SNPs). At such a SNP, there are a total of nine genotypes, *AAAAAAAA* (8*A*), *AAAAAAAa* (7*A*1*a*), *AAAAAAaa* (6*A*2*a*), *AAAAAaaa* (5*A*3*a*), *AAAAaaaa* (4*A*4*a*), *AAAaaaaa* (3*A*5*a*), *AAaaaaaa* (2*A*6*a*), *Aaaaaaaa* (1*A*7*a*), and *aaaaaaaa* (8*a*) in an octoploid population. These genotypes will produce five types of tetraploid gametes during meiosis, *AAAA*, *AAAa AAaa*, *Aaaa*, and *aaaa*, with different frequencies determined by Mendel’s first law and the rate of double reduction (*α*) defined as the probability of two sister chromatids occurring in the same gamete (Darlington 1929; Mather 1935; Haynes and Douches 1993). For 8*A* or 8*a*, the same gamete type is identified although its formation results from either double reduction or non-double reduction. Table 1 tabulates the frequencies of tetraploid gametes produced by each octoploid genotype. We use *AAAAAAAa* (written as *A*_1_ *A*_2_ *A*_3_ *A*_4_ *A*_5_ *A*_6_ *A*_7_ *a* for the identification of allele *A*) as an example to demonstrate how we derive these gamete frequencies. This genotype produces 8 diploids *A*_1_ *A*_1_, *A*_2_ *A*_2_, *A*_3_ *A*_3_, *A*_4_ *A*_4_, *A*_5_ *A*_5_, *A*_6_ *A*_6_, *A*_7_ *A*_7_, *aa*, with the total frequency of *α*, through double reduction, and 28 diploids *A*_1_ *A*_2_, *A*_1_ *A*_3_, *A*_1_ *A*_4_, *A*_1_ *A*_5_, *A*_1_ *A*_6_, *A*_1_ *A*_7_, *A*_1_ *a*, *A*_2_ *A*_3_, *A*_2_ *A*_4_, *A*_2_ *A*_5_, *A*_2_ *A*_6_, *A*_2_ *A*_7_, *A*_2_ *a*, *A*_3_ *A*_4_, *A*_3_ *A*_5_, *A*_3_ *A*_6_, *A*_3_ *A*_7_, *A*_3_ *a*, *A*_4_ *A*_5_, *A*_4_ *A*_6_, *A*_4_ *A*_7_, *A*_4_ *a*, *A*_5_ *A*_6_, *A*_5_ *A*_7_, *A*_5_ *a*, *A*_6_ *A*_7_, *A*_6_ *a*, *A*_7_ *a*, with the total frequency of 1–α, without double reduction. Together, we have the frequencies of *AA* as (1/8)*α*×7 + (1/28)(1–*α*)×21 = 3/4 + 1/8*α*, the frequencies of *Aa* as (1/8)*α*×0 + (1/28)(1–*α*)×7 = 1/4 – 1/4*α*, and the frequencies of *aa* as (1/8)*α*×1 + (1/28)(1–*α*)×0 = 1/8*α*. Tetraploid gametes produced by genotype *AAAAAAAa* include *AAAA*, with frequency calculated as (3/4 + 1/8*α*)^2^ = 9/16 + 1/64α^2^+3/16α, *AAAa*, with frequency as 2(3/4 + 1/8*α*) (1/4 – 1/4*α*) = 3/8−1/16α^2^ −5/16α, *AAaa*, with frequency as 2(3/4 + 1/8*α*)(1/8*α*) + (1/4 – 1/4*α*)^2^ = 1/16 + 3/32α^2^ +1/16α, and *Aaaa*, with frequencies as 2(1/4 – 1/4*α*)(1/8*α*) = (−1/16)α^2^+1/16α, and *aaaa*, with frequency as (1/8*α*)^2^ = 1/64*α* ^2^. All other gamete frequencies can be derived in a similar way.

**TABLE 1:** Gamete frequencies generated by different autooctoploid genotypes.

**Gamete**					
**Genotype**	***AAAA***	***AAAa***	***AAaa***	***Aaaa***	***aaaa***
*AAAAAAAA*	1	0	0	0	0
*AAAAAAAa*	9/16 + 1/64α^2^+3/16α	3/8−1/16α^2^	1/16 + 3/32α^2^ +1/16α	(−1/16)α^2^	1/64α^2^
		−5/16α		+1/16α	
*AAAAAAaa*	225/784 + 9/196α^2^ +45/196α	45/98−9/49α^2^	87/392 + 27/98α^2^	3/98−9/49α^2^	1/784 + 9/196α^2^+3/196α
		−27/98α	−6/49α	+15/98α	
*AAAAAaaa*	25/196 + 225/3136α^2^ +75/392α	75/196	285/784	45/392	9/784
		−225/784α^2^	+675/1568α^2^	−225/784α^2^ +135/784α	+225/3136α^2^+45/784α
		−75/784α	−255/784α		
*AAAAaaaa*	9/196 + 4/49α^2^	12/49−16/49α^2^ +4/49α	41/98 + 24/49α^2^	12/49−16/49α^2^	9/196 + 4/49α^2^+6/49α
	+6/49α		−20/49α	+4/49α	
*AAAaaaaa*	9/784 + 225/3136α^2^ +45/784α	45/392−225/784α^2^ + 135/784α	285/784+	75/196	25/196
			675/1568α^2^	−225/784α^2^−75/784α	+225/3136α^2^+75/392α
			−255/784α		
*AAaaaaaa*	1/784 + 9/196α^2^ +3/196α	3/98−9/49α^2^ + 15/98α	87/392 + 27/98α^2^	45/98−9/49α^2^	225/784
			−6/49α	−27/98α	+9/196α^2^
					+45/196α
*Aaaaaaaa*	1/64α^2^	(−1/16)α^2^	1/16 + 3/32α^2^	3/8−1/16α^2^	9/16
		+1/16α	+1/16α	−5/16α	+1/64α^2^ + 3/16α
*aaaaaaaa*	0	0	0	0	1

The newly-produced gametes combine randomly to generate offspring genotypes. We use *P*_*j*_(*t*) (*j* = 1 for 8*A*, 2 for 7*A*1*a*, 3 for 6*A*2*a*, 4 for 5*A*3*a*, 5 for 4*A*4*a*, 6 for 3*A*5*a*, 7 for 2*A*6*a*, 8 for 1*A*7*a*, and 9 for 8*a*) to denote the frequencies of nine octoploid genotypes in the *t*th (parental) generation. Random mating of these parental genotypes produces 45 possible combination types, each of which forms different offspring genotypes, with frequencies depending on Table 1’s gamete frequencies and the frequencies of 45 parental combinations (Supplementary Table S1). We derive the mathematical expression of each genotype frequency in the (*t*+1)th (offspring) generation derived from those of the *t*th (parental) generation after random mating. Let $P_{j_{1}j_{2}}^{j}\left(t+1\right)$ denote the frequency of offspring genotype j derived from parental mating type $j_{1}\times j_{2}$ ( $j_{1}\leq j_{2}=1,\ldots ,9$ ). Then, the total frequency of offspring genotype *j* is the sum of these offspring genotype frequencies, weighted by mating frequencies, expressed as

$$P_{j}\left(t+1\right)=\overset{9}{\underset{j_{1}\leq j_{2}=1}{\sum }}\left(P_{j_{1}}\left(t\right)\times P_{j_{2}}\left(t\right)\right)P_{j_{1}j_{2}}^{j}\left(t+1\right)$$ (1)

This explicit expression of Eq. 1 is a group of recursive equations, indicating that the frequency of genotype *j* in the (*t*+1)th generation is jointly determined by the mating frequencies of relevant genotypes in the *t*th generation, Mendelian segregation, and double reduction rate. As can be seen from Table S1, *P*_*j*_(*t*+1) has a complicated but explicit relationship with *P*_*j*_(*t*).

**FIGURE 1:**
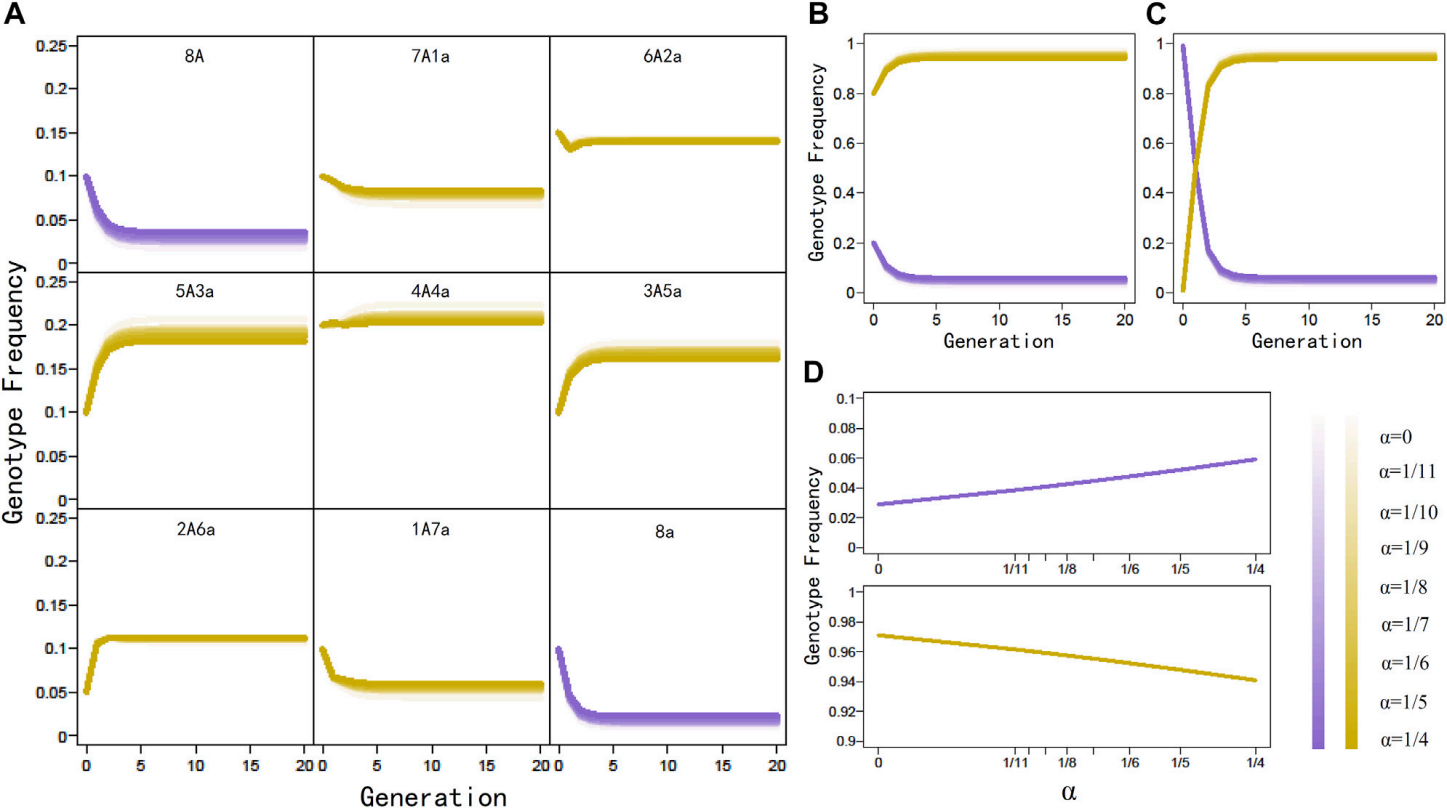
Generation-dependent change of genotype frequencies under different levels of double reduction (*α*) in a panmictic octoploid population. **(A)** Change trends of individual genotype frequencies, initiated with (0.10, 0.10, 0.15, 0.10, 0.20, 0.10, 0.05, 0.10, 0.10). **(B)** Change trends of homozygote and heterozygote genotype frequencies derived from **(A)**. **(C)** Change trends of homozygote and heterozygote genotype frequencies, initiate with extremely high homozygote frequencies (0.99) and extremely low heterozygote frequencies (0.01). **(D)** Equilibrium genotype frequencies of homozygote and heterozygote change as a function of *α*. Legends of *α* are indicated by color metrics.

To numerically explore how *P*_*j*_(*t*) transmits to *P*_*j*_(*t*+1), we randomly sample nine parental genotype frequencies from their space, substitute these sampled values under different double reduction rates into the expression of offspring genotype frequencies given in Eq. 1, use these estimated offspring genotype frequencies to calculate the subsequent offspring genotype frequencies, and repeat this process until 20 generations have passed. By plotting genotype frequencies against generation (Figure 1A), we find that genotype frequencies will not reach absolutely stable values even after many generations of random mating. However, genotype frequencies will quickly approach the stationarity after 15 generations. We repeat the above procedure by sampling 1,000 sets of initial genotype frequencies, from which a similar conclusion is reached, i.e., octoploid genotype frequencies do not attach exact HWE but aHWE over generation, a similar phenomenon detected in tetraploids. Generation 15 can be regarded as one after which the octoploid population is at aHWE.

We plot the overall frequencies of genotypes 8*A* and 8*a* and those of genotypes 7*A*1*a*, 6*A*2*a*, 5*A*3*a*, 4*A*4*a*, 3*A*5*a*, 2*A*6*a*, and 1*A*7*a* over generation (Figure 1B), from which we find that homozygotes and heterozygotes change their frequencies differently during the approximation of aHWE. While homozygote frequency tends to decrease with generation, heterozygote frequency displays a generation-dependent increase. In an extreme example, we start the homozygote frequency of near one and the heterozygote frequency of near zero as initial values, in which the same trends are observed (Figure 1C). This phenomenon suggests that by approaching aHWE, octoploids are equipped to increase genetic diversity in natural populations.

We also investigate how double reduction influences the approximation of aHWE in octoploids. Under a range of double reduction rates, we find that octoploids are always close to stabilize their genotype frequencies in 15 generations, suggesting that double reduction is neutral for aHWE (Figure 1A). The case of no double reduction in the autopolyploid model reduces to allopolyploids if no preferential pairing is assumed. Thus, it is postulated that both autopolyploids and allopolyploids follow the same rule of aHWE. In autooctoploids, double reduction does influence the distribution of equilibrium genotype frequencies to some extent; the frequency of double reduction is positively associated with equilibrium frequencies of homozygotes but negatively associated with the equilibrium frequencies of heterozygotes (Figure 1D). Thus, double reduction may be regarded as a determinant of genetic diversity in autopolyploids.

## Statistical Testing of aHWE

### Recursive Test

This test was proposed by Sun et al. (2020) to test aHWE in tetraploids. It is straightforward to extend it to test octoploid aHWE. As shown from recursive Eq. 1, an octoploid population reaches asymptotic equilibrium at approximately generation 15 (Figure 1A). Thus, the genotype frequencies at generation 15 are regarded as a proxy of equilibrium frequencies, denoted as ${\bar{P}}_{j}$ for a SNP. Let *N*_*j*_ (totaling to *N*) and *P*_*j*_ denote the observations and frequencies of nine genotypes in the current octoploid natural population. We formulate the likelihood of genotypic observations as

$$L\left(N\right)=c+\overset{9}{\underset{j=1}{\sum }}N_{j}\log\left(P_{j}\right)$$ (2)

where *c* is the constant and the maximum likelihood estimates (MLEs) of *P*_*j*_ can be solved as ${\hat{P}}_{j}=N_{j}/N$ . We test whether equilibrium genotype frequencies are not different from observed genotype frequencies by using Pearson’s chi-square testing approach, expressed as

$$\chi^{2}=N\overset{9}{\underset{j=1}{\sum }}\frac{{\left({\hat{P}}_{j}-{\bar{P}}_{j}\right)}^{2}}{{\bar{P}}_{j}}$$ (3)

which is thought to follow a chi-square distribution with eight degrees of freedom. If test statistics $\chi^{2}$ is larger than the critical value $\chi_{95\%,8}^{2}$ , then we claim that the population deviates from aHWE at the SNP considered. Otherwise, the population is at equilibrium for this locus. Multiple comparisons will be needed if a set of SNPs are tested in a population genetic study.

### Gamete-Based Model

Under HWE, genotype frequencies are expressed as the frequency products of the gametes that form the genotypes. Let *P*_*AAAA*_, *P*_*AAAa*_, *P*_*AAaa*_, *P*_*Aaaa*_, and *P*_*aaaa*_ denote the frequencies of five tetraploid gametes produced by octoploid genotypes in a natural population. Equilibrium genotype frequencies (*Q*_*j*_) are expressed as

$$\begin{array}{l} Q_{8A}=P_{AAAA}^{2}\\ Q_{7A1a}=2P_{AAAA}P_{AAAa}\\ Q_{6A2a}=2P_{AAAA}P_{AAaa}+P_{AAAa}^{2}\\ Q_{5A3a}=2P_{AAAA}P_{Aaaa}+2P_{AAAa}P_{AAaa}\\ Q_{4A4a}=2P_{AAAA}P_{aaaa}+2P_{AAAa}P_{Aaaa}+P_{AAaa}^{2}\\ Q_{3A5a}=2P_{AAAa}P_{aaaa}+2P_{AAaa}P_{Aaaa}\\ Q_{2A6a}=2P_{AAaa}P_{aaaa}+P_{Aaaa}^{2}\\ Q_{1A7a}=2P_{Aaaa}P_{aaaa}\\ Q_{8a}=P_{aaaa}^{2} \end{array}$$ (4)

We formulate a likelihood under HWE, i.e.,

$$L_{g}\left(N\right)=c+\overset{9}{\underset{j=1}{\sum }}N_{j}\log\left(Q_{j}\right)$$ (5)

which includes multiple mixture terms with component proportions determined by gamete frequencies. We implement the derivative-free EM algorithm to obtain the MLEs of gamete frequencies ( ${\hat{Q}}_{j}$ ) under HWE. Plugging the ${\hat{Q}}_{j}$ values into $L_{g}\left(N\right)$ (4) obtains the likelihood value under the null hypothesis that the population is at HWE. The likelihood under the alternative hypothesis, i.e., the population deviates from HWE, is calculated by Eq. 2. The log-likelihood ratio (LR) is then calculated as

$$LR_{g}=-2\left(L_{g}\left(N\right)-L\left(N\right)\right)$$ (6)

which follows a chi-square distribution with four degrees of freedom. By comparing the LR test statistics with critical value $\chi_{95\%,4}^{2}$ , we determine whether the SNP considered is at HWE in the octoploid population.

### Allele-Based Model

Because of technical and economic reasons, many studies may genotype octoploids at a limited resolution, in which case seven heterozygotes 7A1a, 6A2a, 5A3a, 4A4a, 5A3a, 6A2a, and 7A1a cannot be distinguished from each other. Thus, for such dosage-unknown markers, there are only three distinguishable genotypes, i.e., two homozygotes each for an alternative allele and one mixed heterozygote. The frequencies and sizes of two homozygotes and the heterozygote at a dosage-unknown SNP are denoted as *P*_8A_, *P*_8a_, and *P*_ and *N*_8A_, *N*_8a_, and *N*_, respectively. It is impossible that five gamete frequencies are estimated from three distinguishable genotypes. To make HWE testable for dosage-unknown markers, we make an assumption; i.e., alleles randomly unite to generate gametes during meiosis. Let *p* and *q* denote allele frequencies of *A* and *a*, respectively. Under HWE, we express genotype frequencies of homozygotes and heterozygote in terms of allele frequencies, i.e.,

$$\begin{array}{l} Q_{8A}=p^{8}\\ Q_{\_\_}=8p^{7}q+28p^{6}q^{2}+56p^{5}q^{3}+70p^{4}q^{4}+56p^{3}q^{5}+28p^{2}q^{6}+8pq^{7}\\ Q_{8a}=q^{8} \end{array}$$ (7)

Under the null hypothesis of HWE, the likelihood is formulated as

$$L_{e}\left(N\right)=c+N_{8A}\log\left(Q_{8A}\right)+N\_\log\left(Q_{\_}\right)+N_{8a}\log\left(Q_{8a}\right)$$ (8)

in a comparison with the likelihood under the alternative hypothesis of no HWE,

$$L\left(N\right)=c+N_{8A}\log\left(P_{8A}\right)+N\_\log\left(P_{\_}\right)+N_{8a}\log\left(P_{8a}\right)$$ (9)

Likelihood Eq. 8 contains a mixture term, whose solution can be made by implementing the derivative-free EM algorithm. We calculate the LR from likelihoods Eqs 8, 9 as a test statistic to test whether the dosage-unknown SNP considered is at HWE. This test statistic is thought of as being following a chi-square distribution with one degree of freedom.

## Results

### Example

In a genome-wide association study of allopolyploid switchgrass, Grabowski et al. (2017) collected samples from the southern-adapted upland ecotype and northern-adapted upland ecotype of this species in the United States. The sampled plants include allotetraploids from both ecotypes and allooctoploids from the upland ecotype. Here, we test whether upland octoploids, distributed in the east and west regions, deviate from aHWE. East and west populations include 66 and 101 samples, for which 24,859 and 23,795 quality SNPs are, respectively, available for our analysis. All markers are dosage-unknown, at each of which there are three distinguishable genotypes. Thus, the allele-based model is used to test whether they are at aHWE in the populations.

**FIGURE 2:**
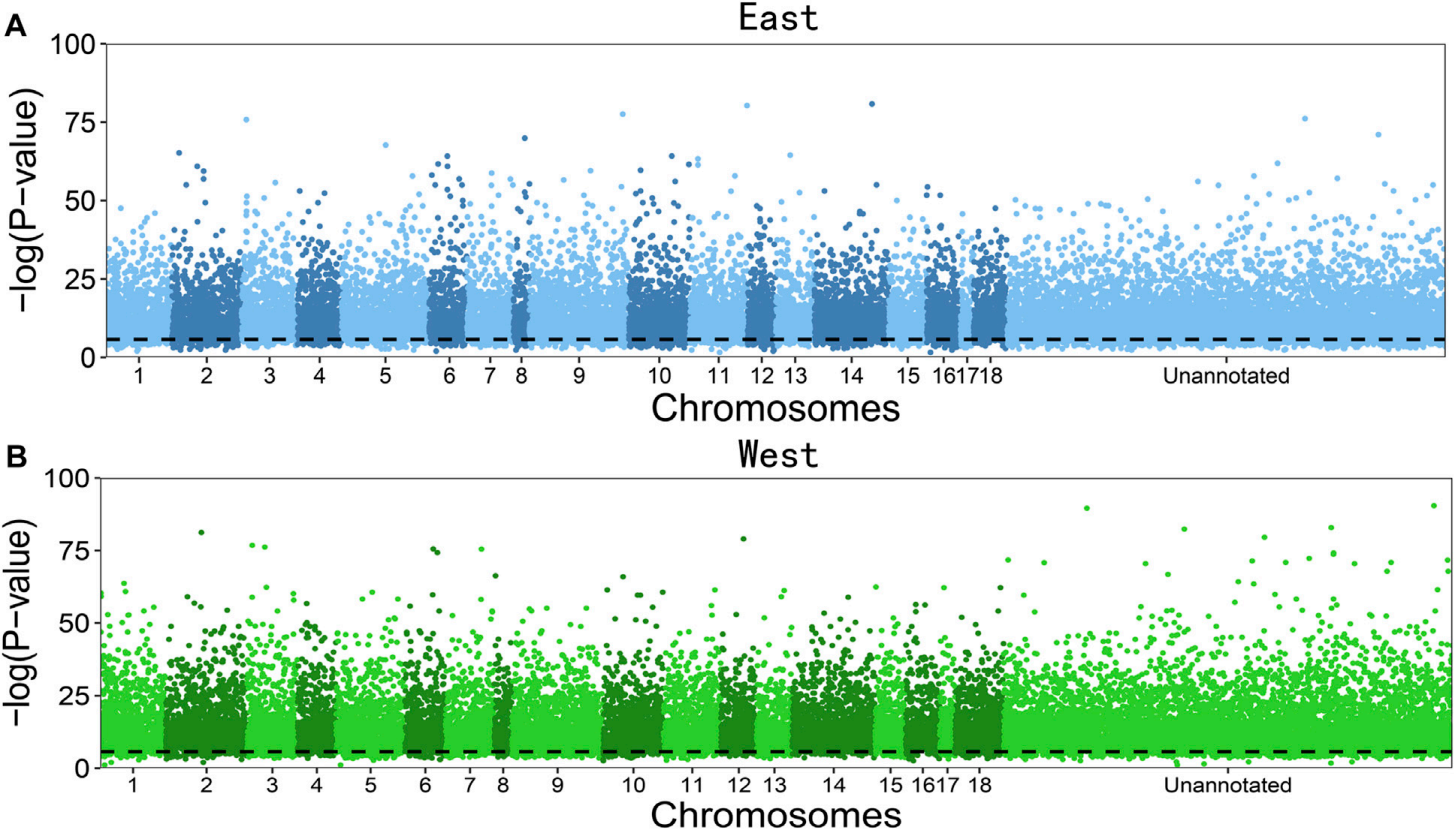
Manhattan plots of significance test for marker aHWE throughout the genome in an allooctoploid switchgrass upland ecotype collected from eastern **(A)** and western populations **(B)**. SNPs with unknown chromosomes are given in the “unannotated” part. Horizontal line denotes the significance level after Bonferroni correction.

Figure 2 illustrates the significance test of deviation from aHWE for dosage-unknown SNPs distributed throughout the switchgrass genome in the east and west populations of the upland ecotype. The majority of SNPs are detected to deviate from aHWE, with the western population having a slightly larger proportion (93.3%) than the eastern population (90.7%). This result suggests that the segregating genes of allootoploid switchgrass plants, especially those from the western population, throughout the entire genome are on their way toward equilibrium. In a similar aHWE test for allotetraploids of the same species, only a small proportion of SNPs was detected to deviate from aHWE (Sun et al., 2020). This comparison suggests that while lower-ploidy switchgrass tends to be evolutionarily stable, higher-ploidy switchgrass is still experiencing a strong evolutionary change.

### Power Analysis

We perform simulation studies to examine the power of HWE detection. Using given gamete frequencies for dosage-known markers and allele frequencies for dosage-unknown markers, we simulate genotype data by allowing simulated genotype frequencies to deviate from equilibrium genotype frequencies by 20%. Under this deviation, genotype data are simulated for different sample sizes (*N* = 50, 100, 200, 400). We use the gamete- and allele-based models to test HWE for dosage-known and dosage-unknown markers, respectively. This simulation and estimation process is repeated 1,000 times to empirically calculate HWE detection power, defined as the proportion of the simulations for which LR tests are significant.

We find that to detect aHWE for octoploid populations, a sample size of *n* = 200 is required, under which the power of nonequilibrium detection reaches 0.94 (Table 2). A sample size of *n* = 100 can only have about 0.60 power, whereas *n* = 50 fails to detect deviation from aHWE in most cases. We also analyze the false positive rate of the model by simulating the data with genotype frequencies deviating from equilibrium genotype frequencies by zero. In all cases, the model has a low type I error (Table 2).

**TABLE 2:** Power analysis and false positive rate of aHWE detection under different sample size.

Degree of deviation	50	100	200	400
0	0.03	0.02	0.01	0.01
20	0.37	0.58	0.94	1.00

Note: Degree of deviation describes how much genotypes frequencies deviate from equilibrium frequencies.

## Discussion

It has been recognized that polyploids gradually reach HWE through random mating, but it is not very clear how many generations they mate to approach equilibrium (Geiringer 1949; Bever and Felber 1992). Through a mathematical derivation, we showed that tetraploids never attain absolute HWE but aHWE after 5 generations of random mating (Sun et al., 2020). This phenomenon, in contrast to diploids in which exact HWE can be achieved only after one generation of random mating, suggests that polyploids are a “perpetual machine” of evolution; i.e., they can evolve even without actions of evolutionary forces, such as natural selection, mutation, genetic drift, admixture and so on.

In this article, we show that octoploids require more generations (i.e., 15) to approach aHWE. As compared to low ploidy-level polyploids, high ploidy-level polyploids have more allelic combinations and, thus, larger genetic diversity (Johnson and Vance-Borland, 2016; Grabowski et al., 2017). More generations required to reach aHWE suggest that high ploidy-level polyploids can maintain a longer-standing time of evolution than low ploidy-level polyploids. This theoretical postulation is well in agreement with empirical observations for many plants whose high-ploidy polyploids have greater diversity than low-ploidy relatives in the same habitat (Johnson and Vance-Borland, 2016). Double reduction is an important phenomenon in autopolyploids. It has been thought to affect evolutionary processes due to genetic drift (Moody et al., 1993) and gametophytic selection (Butruille and Boiteux 2000). Double reduction can change population structure and diversity by increasing the frequencies of homozygotes, but it does not strikingly accelerate the evolution of autopolyploid populations because of its subtle impact on deviation from HWE.

Based on mathematical derivations of frequency transmission, we propose three approaches for testing aHWE in octoploids using dosage-known and dosage-unknown markers. By analyzing dosage-unknown marker data of octoploid switchgrass collected from its natural distribution (Grabowski et al., 2017), we validate the usefulness of the gamete-based equilibrium-detecting model. Computer simulation is performed to determine the sample size required to detect aHWE, as guidance for designing octoploid population and evolutionary genetic studies. An increasing number of studies have begun to investigate the ecological relationship between HWE deviation and the organisms’ adaptation to heterogeneous environments (Tyukmaeva et al., 2011; Tarazona et al., 2019; Carvalho et al., 2021). Our aHWE testing model provide a generic tool to infer the genetic variation and evolutionary status of octoploid populations.

## Data Availability

The data and code used in this article can be freely downloaded at https://github.com/CCBBeijing/OctoploidDeer.
